# Fegeler Syndrome Mimicking Kaposi Sarcoma: A Case Report

**DOI:** 10.7759/cureus.71902

**Published:** 2024-10-20

**Authors:** Kerem Balan, Başak Yalıcı Armağan, Berkercan Tus, Özay Gököz

**Affiliations:** 1 Dermatology, Hacettepe University, Ankara, TUR; 2 Pathology, Hacettepe University, Ankara, TUR

**Keywords:** acquired capillary malformation, dermoscopic features, fegeler syndrome, kaposi sarcoma, nodular vascular lesions

## Abstract

Acquired capillary malformation, also known as Fegeler syndrome, is a rare condition in adults, often linked to trauma. This report presents a 49-year-old male with a violaceous nodule on the fourth toe, clinically resembling Kaposi’s sarcoma. Dermoscopic examination revealed surface scaling, thick intersecting white lines, and a rainbow pattern. Despite the clinical and dermoscopic similarities to Kaposi’s sarcoma, histopathological evaluation confirmed a diagnosis of capillary malformation. The patient had no history of significant trauma or medication use, though minor trauma from foot placement may have contributed. This case emphasizes the importance of considering capillary malformation in the differential diagnosis of nodular vascular lesions on the foot.

## Introduction

Capillary malformations consist of dermal capillaries and postcapillary venules [1]. Acquired capillary malformation (also known as Fegeler Syndrome) is rarer than the congenital form [2]. Acquired form most often occurs in adults following trauma. Herein, we present a rare case of acquired capillary malformation on the toe, which mimics Kaposi sarcoma.

## Case presentation

**Figure 1:**
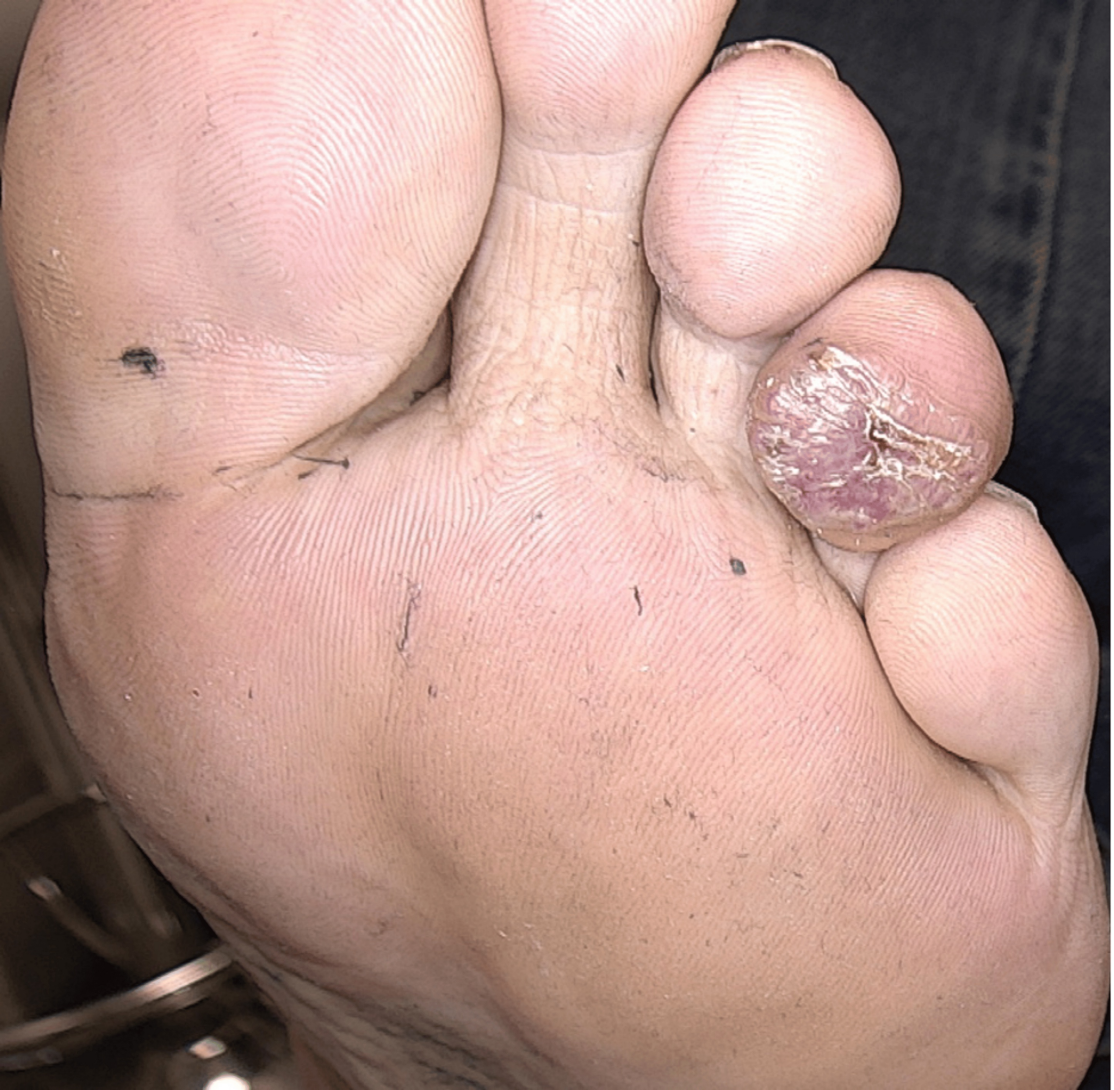
A hyperkeratotic violaceous nodule with hemorrhagic crusts on the fourth toe of the left foot.

**Figure 2:**
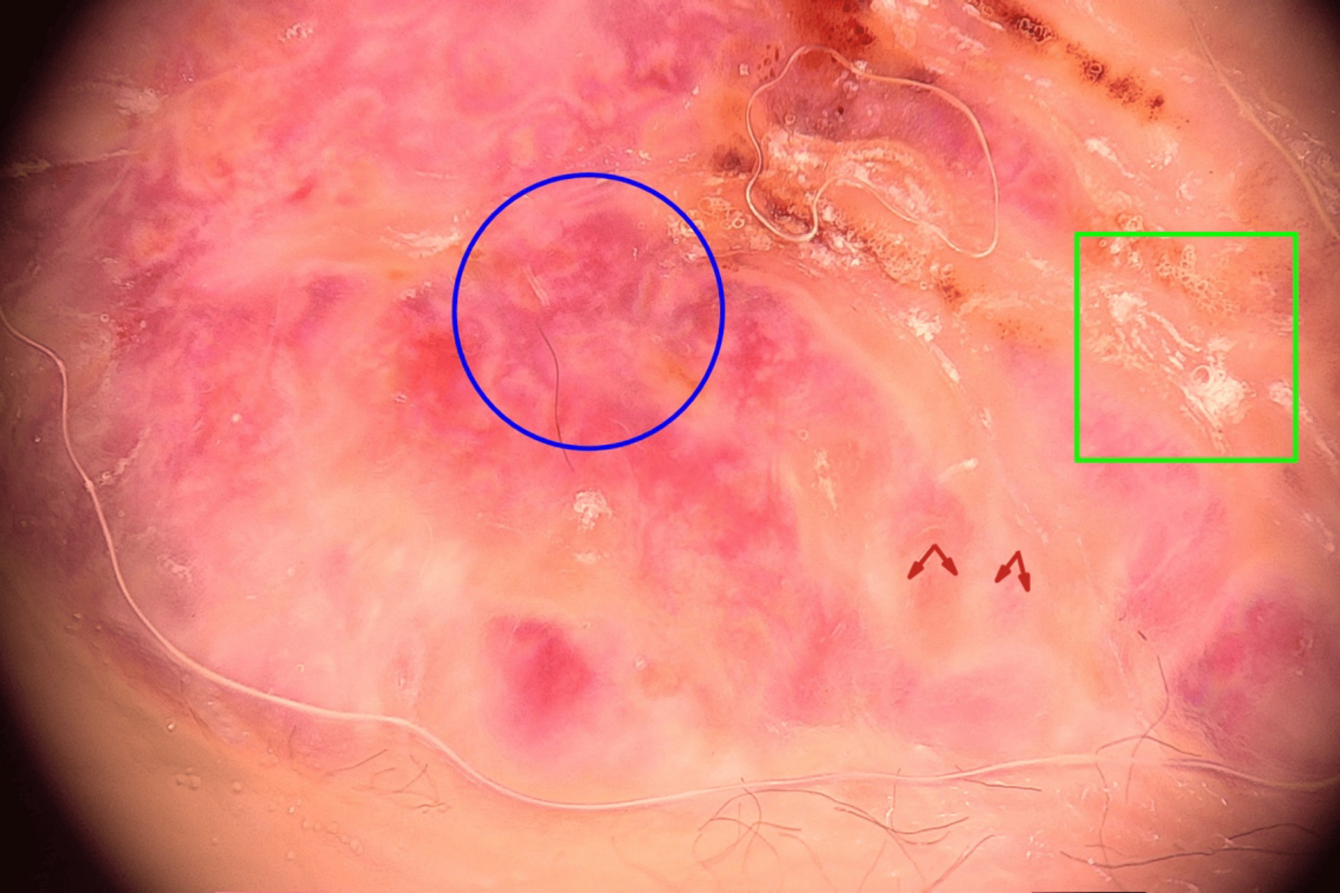
Dermoscopic features; rainbow pattern (blue circle); surface scaling (yellow square); intersecting thick white lines (red arrows)

**Figure 3:**
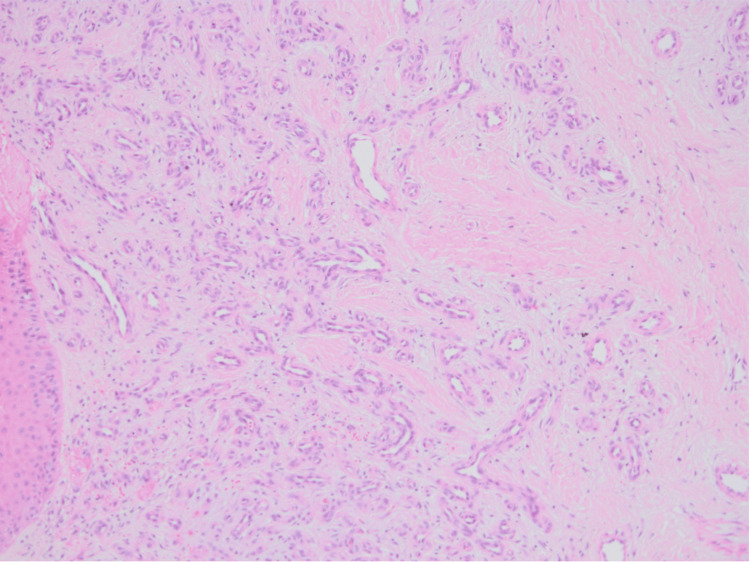
Capillaries and fewer arteriolar structures proliferate between collagen bundles (H.E. x100)

A 49-year-old male patient presented with a lesion on his toe for one year. He denied any history of trauma. He does not have any comorbidities or medication use. A dermatological examination revealed a violaceous nodule was detected on the plantar surface of the left fourth toe (Figure 1). In dermoscopic examination, surface scaling, intersecting thick white lines, and polychromatic color changes (rainbow pattern) were identified (Figure 2). Histopathological examination of punch biopsy material revealed capillary malformation. However, the entire lesion was excised to rule out Kaposi sarcoma or amelanotic malign melanoma. Excisional biopsy was also reported as a capillary malformation. On histopathological examination, a dermal capillary proliferation was seen. The prominent lobular architecture was not present. Some of the capillaries demonstrated a thickened wall (Figure 3). Herpes Virus Type 8 (HHV-8) was negative. Following the total excision of the lesion, no new lesion development or recurrence was observed during follow-up.

## Discussion

Capillary malformation is typically observed in childhood and is quite rare in adulthood [3]. Trauma and use of isotretinoin or statin group drugs were identified as contributing factors for adult-onset form [1]. Although there was no medication use or major trauma in our case, the lesion may have developed due to minor traumas related to foot placement. In the literature, cases of acquired capillary malformation are frequently reported as pink to purpule patches rather than nodules [1-3]. The current case is presented because of its nodular form and similarity to Kaposi sarcoma. Kaposi sarcoma typically manifests with violaceous plaques and nodules in the lower extremities [4]. Dermoscopic characteristics of Kaposi sarcoma are white lines and clods, surface scaling, a rainbow pattern, four dot clods, and a collarette sign. Although the clinical and dermoscopic features were similar to Kaposi sarcoma, histopathological examination confirmed the diagnosis of capillary malformation. While the literature generally describes lesions as patch-like, nodular lesions, such as in our case, can be particularly challenging and may lead to diagnostic confusion. The literature emphasizes the potential benefits of laser treatment, especially pulsed dye laser, and in certain cases, particularly for patients who decline treatment, simple monitoring may also be appropriate [1,5].

## Conclusions

Acquired capillary malformation, although typically rare in adults, can present as a diagnostic challenge due to its clinical resemblance to conditions like Kaposi’s sarcoma. In this case, the patient presented with a violaceous nodule on the toe, mimicking Kaposi’s sarcoma both clinically and dermoscopically. However, histopathological examination confirmed the diagnosis of capillary malformation. Despite the absence of significant trauma or medication use, minor trauma may have played a role in the lesion’s development. Dermatologists should keep capillary malformation in mind in the differential diagnosis of kaposi sarcoma in nodular vascular lesions of the foot.
